# Right-Hemisphere Language Dominance Unmasked by Acute Ischemic Stroke: A Case Report

**DOI:** 10.7759/cureus.104995

**Published:** 2026-03-10

**Authors:** Carlos Dieguez-Campa, Daniela González-Sangabriel, Rebeca de Jesus Ramos-Sánchez, Kevin G Enriquez Peregrino, Raúl Medina-Rioja

**Affiliations:** 1 Neurology Department, Instituto Nacional de Neurología y Neurocirugía "Manuel Velasco Suárez", Mexico City, MEX; 2 Psychiatry Department, Instituto Nacional de Neurología y Neurocirugía "Manuel Velasco Suárez", Mexico City, MEX; 3 Neuroradiology Department, Instituto Nacional de Neurología y Neurocirugía "Manuel Velasco Suárez", Mexico City, MEX; 4 Stroke Clinic, Instituto Nacional de Neurología y Neurocirugía "Manuel Velasco Suárez", Mexico City, MEX; 5 Cognitive Neurology and Dementia Clinic, Instituto Nacional de Neurología y Neurocirugía "Manuel Velasco Suárez", Mexico City, MEX

**Keywords:** broca's aphasia, crossed aphasia, dextrals, motor aphasia, stroke

## Abstract

Language functions are typically lateralized to the left cerebral hemisphere in most right-handed individuals; however, a minority exhibit atypical language dominance, including right-hemisphere representation. In these cases, right-hemispheric lesions may result in aphasia, a rare entity known as crossed aphasia in dextrals (CAD). We report a 63-year-old strongly right-handed individual with no familial history of left-handedness who developed severe motor aphasia following an acute right-hemispheric ischemic stroke.

The patient presented with a sudden-onset language disturbance and left-sided weakness. Neurological examination revealed severe non-fluent aphasia with impaired speech production and repetition, preserved comprehension, and left hemiparesis, with a National Institutes of Health Stroke Scale (NIHSS) score of 14. Initial CT scan demonstrated an acute infarction in the right middle cerebral artery territory without left-hemispheric involvement. Etiologic evaluation identified critical aortic stenosis, suggesting a probable cardioembolic mechanism. Neuropsychological assessment confirmed severe motor aphasia secondary to a right-hemispheric lesion. Motor deficits improved during hospitalization; however, expressive aphasia persisted and required intensive speech therapy.

This case highlights the variability of cerebral language organization and the limitations of inferring language dominance solely from handedness. It supports network-based models of language that emphasize the contribution of the right-hemisphere and subcortical structures to expressive language function. Clinically, systematic language assessment in right-hemispheric stroke and early targeted rehabilitation are essential to optimize outcomes in patients with atypical language lateralization.

## Introduction

Language dominance is typically localized to the left cerebral hemisphere in approximately 90% of right-handed individuals [1]. However, a small percentage of individuals (1%-13% of all patients), including some dextrals, exhibit atypical language lateralization, where language functions are either localized to the right hemisphere or distributed bilaterally [2]. This atypical dominance can manifest as an entire right-hemisphere lateralization or as dissociated language functions, where expressive or receptive language components are differentially localized [3]. In cases of right-hemisphere dominance, lesions in the right hemisphere can precipitate aphasia, a condition known as crossed aphasia in dextrals (CAD). This phenomenon is particularly rare in dextral individuals, occurring in about 30% of sinistrals but very infrequently in those with a strong right-hand preference without a familial history of left-handedness [4]. Our case report details a 63-year-old right-handed individual with no familial history of left-handedness who experienced a right-sided ischemic stroke, leading to severe classic motor aphasia (Broca's).

## Case presentation

A 63-year-old Mexican man presented to the emergency department with sudden-onset language disturbance and left-sided weakness. Past medical history was notable for poorly controlled type 2 diabetes mellitus.

Given the suspicion of acute stroke, the National Institutes of Health Stroke Scale (NIHSS) score was assessed, yielding 14 points, driven by left-sided hemiparesis, gaze deviation to the right, sensation abnormalities, and language impairment. Detailed neurological examination revealed severe motor aphasia, characterized by marked difficulty in speech production and impaired repetition, accompanied by left-sided hemiparesis. The comprehension of spoken language was preserved, consistent with a selective impairment of expressive language functions.

Due to the acute onset, lack of fluctuations, and preserved consciousness, delirium and apraxia of speech were considered unlikely. Moreover, unlike pure apraxia of speech, this case presented deficits in language structure and repetition that extended beyond simple motor planning. The greater awareness of crossed aphasia may improve diagnostic accuracy, prognostication, and rehabilitation strategies in patients with atypical language lateralization.

**Figure 1:**
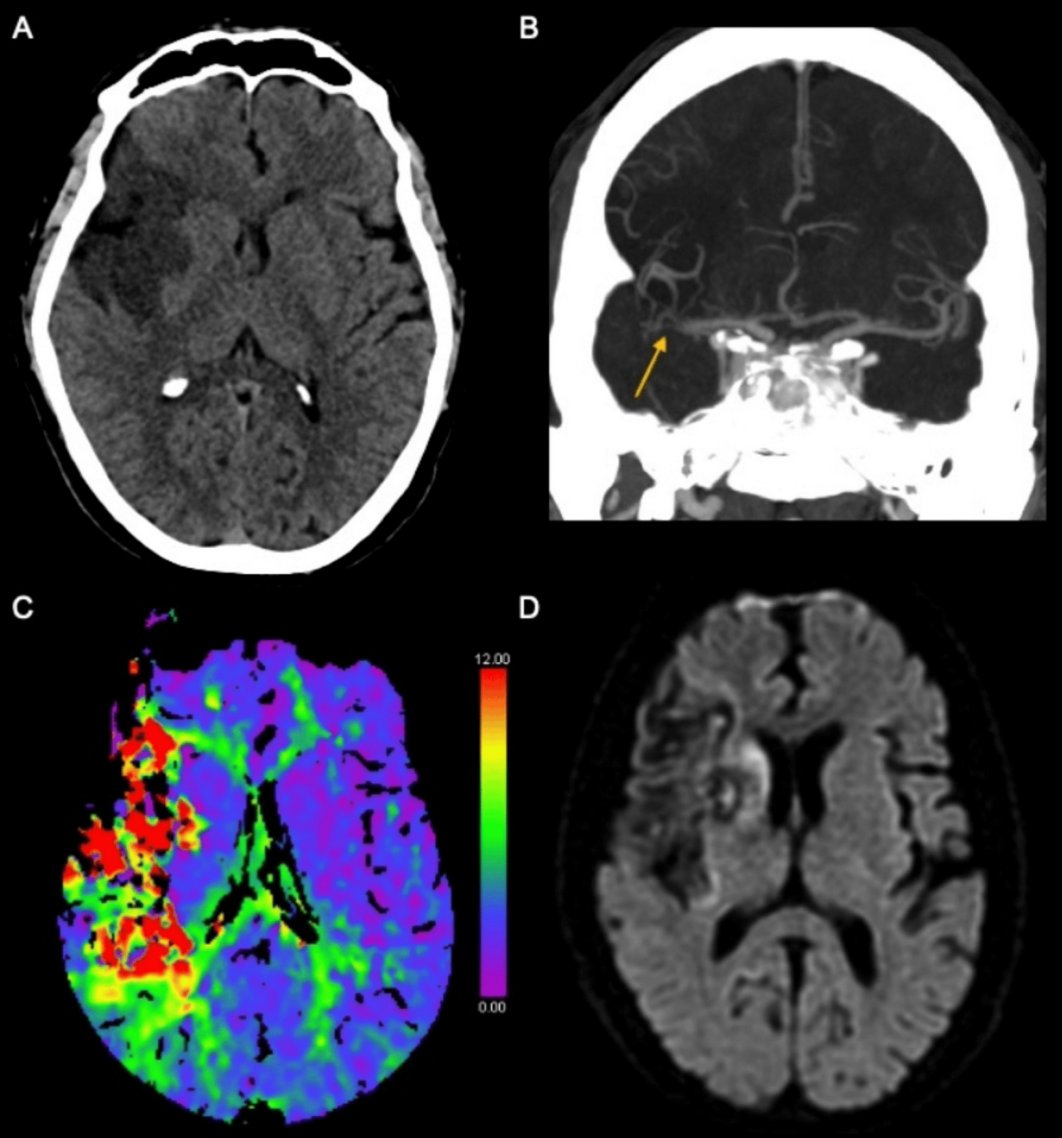
(A) Noncontrast CT shows a large hypodense infarct in the right MCA territory. (B) Coronal CT angiography demonstrates reduced caliber of the distal right M1 segment (arrow). (C) Color-coded Tmax map shows delayed perfusion within the right MCA territory without hypoperfusion in the contralateral hemisphere. (D) Diffusion-weighted imaging obtained one month later shows subacute infarction in the right MCA territory, with no diffusion abnormalities in the left hemisphere MCA: middle cerebral artery

On arrival, the door-to-CT time was five minutes. Multimodal CT imaging demonstrated an acute right-hemispheric infarction involving the middle cerebral artery territory, with an Alberta Stroke Program Early CT Score (ASPECTS) of 3. CT perfusion analysis revealed no evidence of salvageable penumbral tissue, consistent with a large established infarct core. Brain magnetic resonance imaging (MRI) did not reveal additional areas of acute ischemia (Figure 1).

Given the time from symptom onset and the absence of large vessel occlusion, the patient was not a candidate for intravenous thrombolysis or mechanical thrombectomy. He was admitted for further etiologic workup.

Initial Holter monitoring, electrocardiogram, and carotid ultrasound were unremarkable. Transthoracic echocardiography revealed critical aortic stenosis with proximal ascending aortic dilatation, after which the patient was referred to cardiology for further management. Neuropsychological assessment (which included the Barcelona language assessment and the Edinburgh Handedness Inventory) demonstrated crossed aphasia secondary to a right-hemispheric lesion, a rare presentation, as language impairments typically result from left-hemispheric strokes, whereas right-hemispheric lesions are more commonly associated with visuospatial and attentional deficits.

Over the following days, the patient showed improvement in motor function; however, aphasia persisted (Video 1). He was ultimately discharged on aspirin and atorvastatin and enrolled in an intensive speech therapy program to address the persistent motor aphasia, which remained a significant functional deficit despite motor recovery [5]. Although the final stroke etiology could not be definitively established, the presence of significant cardiac abnormalities suggested a cardioembolic mechanism as the most likely cause. However, in the absence of a strong indication for anticoagulation, the patient was started on antiplatelet therapy with aspirin for secondary stroke prevention. The patient remains under close clinical follow-up, with a comprehensive cardiological evaluation pending.

**Video 1:** Language examination reveals impaired fluency with preserved comprehension

## Discussion

Language relies on a distributed yet highly organized network of cortical regions and white-matter pathways, predominantly within the left hemisphere, that support sound-meaning mapping, syntax, semantics, and speech production. Contemporary models have moved beyond the classic Broca-Wernicke framework toward network-based, dual-stream, and multidimensional accounts of language organization. Contemporary models conceptualize this system not as a set of isolated centers but as a functionally coherent, modality-independent network whose defining property is specialization rather than mere anatomical location. Within this framework, hemispheric dominance reflects an emergent property of large-scale network organization rather than a rigid structural rule [6].

In most individuals, language functions are lateralized to the left cerebral hemisphere, whereas the right hemisphere has traditionally been associated with prosody, pragmatics, and discourse-level processing. This hemispheric specialization is thought to arise from structural and connectivity advantages of the left hemisphere: left auditory and language cortices are typically thinner, more extensive, and more highly myelinated (particularly within Heschl's gyrus and the posterior portion of Broca's area), facilitating the faster and more efficient processing of rapidly changing speech sounds. In parallel, the arcuate fasciculus and related frontotemporal white-matter tracts exhibit greater volume and higher microstructural integrity on the left, supporting a more efficient auditory-motor language loop [7-10]. However, accumulating lesion and functional imaging evidence has increasingly challenged a strictly left-lateralized model, demonstrating that right-hemisphere damage after stroke can impair core aspects of language processing, including auditory sentence comprehension, particularly for semantically complex or reversible constructions [11].

CAD refers to the occurrence of aphasia following a right-hemisphere lesion in a right-handed individual, in the absence of early brain injury and without the structural involvement of the left hemisphere. Clinically, CAD may manifest as either fluent or non-fluent syndromes that closely resemble classic left-hemisphere aphasias, despite arising from right-sided lesions and often being accompanied by left hemiplegia. Several mechanisms have been proposed to explain this phenomenon. These include developmental variation resulting in atypical right-hemisphere language dominance, dissociation between handedness and language lateralization (where behaviorally right-handed individuals may harbor a right-hemisphere language network), and the contribution of right perisylvian and subcortical structures, such as the internal capsule, basal ganglia, and frontal or temporal homologues, to language organization [12-15].

In the present case, the involvement of the right lentiform nucleus, a deep subcortical structure, supports prior observations implicating subcortical-cortical network disruption in the pathophysiology of crossed aphasia. The diagnosis of crossed aphasia was considered clinically plausible based on the presence of a right-hemisphere lesion, a congruent aphasic phenotype, and the absence of the structural involvement of the left hemisphere on neuroimaging. Language dominance was inferred clinically, as no formal lateralization testing, such as functional MRI, Wada testing, or diffusion tractography, was performed. We acknowledge that the lack of an objective language lateralization assessment represents a methodological limitation and should be considered when interpreting the findings.

## Conclusions

This case highlights crossed aphasia in a strongly right-handed individual without a familial history of left-handedness, underscoring the variability of cerebral language organization and the limitations of assuming left-hemisphere dominance based solely on handedness. Our findings reinforce the concept of language as a distributed and partially bilateral network, in which right-hemisphere and subcortical structures may play a critical role in expressive language function.

Clinically, this case emphasizes the importance of systematic language assessment in right-hemisphere strokes and supports early, targeted speech therapy when aphasia is identified. The greater awareness of crossed aphasia may improve diagnostic accuracy, prognostication, and rehabilitation strategies in patients with atypical language lateralization. However, the absence of formal language lateralization studies, such as functional MRI or Wada testing, represents a limitation and precludes the definitive confirmation of right-hemisphere language dominance.
